# Few-shot pulse wave contour classification based on multi-scale feature extraction

**DOI:** 10.1038/s41598-021-83134-y

**Published:** 2021-02-12

**Authors:** Peng Lu, Chao Liu, Xiaobo Mao, Yvping Zhao, Hanzhang Wang, Hongpo Zhang, Lili Guo

**Affiliations:** 1 School of Electrical Engineering, Zhengzhou University, Zhengzhou, 450001 China; 2 Research Center for Intelligent Science and Engineering Technology of TCM, Zhengzhou, 450001 China; 3 China Academy of Chinese Medical Sciences, Beijing, 100000 China; 4 Internet Medical and Health Service Henan Collaborative Innovation Center, Zhengzhou, 450001 China; 5 The First Affiliated Hospital of Zhengzhou University, Zhengzhou, 450001 China; 6 First Affiliated Hospital of Shihezi University Medical College, Shihezi, 832000 China

**Keywords:** Biomedical engineering, Cardiovascular diseases, Computer science

## Abstract

The annotation procedure of pulse wave contour (PWC) is expensive and time-consuming, thereby hindering the formation of large-scale datasets to match the requirements of deep learning. To obtain better results under the condition of few-shot PWC, a small-parameter unit structure and a multi-scale feature-extraction model are proposed. In the small-parameter unit structure, information of adjacent cells is transmitted through state variables. Simultaneously, a forgetting gate is used to update the information and retain long-term dependence of PWC in the form of unit series. The multi-scale feature-extraction model is an integrated model containing three parts. Convolution neural networks are used to extract spatial features of single-period PWC and rhythm features of multi-period PWC. Recursive neural networks are used to retain the long-term dependence features of PWC. Finally, an inference layer is used for classification through extracted features. Classification experiments of cardiovascular diseases are performed on photoplethysmography dataset and continuous non-invasive blood pressure dataset. Results show that the classification accuracy of the multi-scale feature-extraction model on the two datasets respectively can reach 80% and 96%, respectively.

## Introduction

The collection process of pulse wave signals is simple and non-invasive, enabling convenient acquisition of PWC data^1–3^. The classification of PWC can be used to monitor the heart condition. However, no uniform standard exists for marking PWC^4^. The standard diagnostics of PWC is primarily based on an individual’s different clinical knowledge and experience to define the morphological features. The formed diagnostic results are subjective, which are difficult to be recognized by all experts.

Existing classification methods of PWC can be categorized into two: statistical methods and machine-learning methods^5,6^. Statistical methods represented by artificial time-frequency domain feature extraction are suitable for stationary sequences^7^. Machine-learning methods represented by convolution neural networks^8,9^ (CNN), recursive neural networks^10,11^ (RNN), and support vector machines^12,13^ are suitable for complex non-linear sequences. Clinically collected PWCs are non-stationary and time varying^14^, rendering it suitable for machine-learning methods.

Long short-term memory (LSTM), a variant of RNN, has the ability of mining long-distance time-series data information^15^. It is extensively used in machine translation^16,17^, fault diagnosis^18,19^, speech recognition^20,21^, and electrocardiogram classification^22,23^. In literature^24^, the representation of speech signals from an original network is automatically learned by CNN, and then the temporal representation of features is learned by LSTM; In literature^25^, the features of wearable sensor data is learned by CNN, and then the time dependence between actions are modeled by LSTM.

The common idea of the above methods is to extract high-dimensional features by CNN and subsequently obtain short-series features through LSTM, which can be used to predict and synthesize time series. PWC is a kind of few-shot data that difficultly meets the training needs of complex deep networks^26^.

In the present study, a recursive-network unit structure based on LSTM is designed and found to be suitable for few-shot PWC. Compared with LSTM structure, it has fewer parameters and faster training rate. Adjacent unit information is transmitted through state variables, information is updated through a forgetting gate, and the long-term dependence of PWC is retained in the form of unit series. Simultaneously, a multi-scale feature-extraction model adapted to few-shot PWC is proposed. The multi-scale feature-extraction model is an integrated model that extracts features from three angles. The spatial features of single-period PWC and the rhythm features of multi-period PWC are extracted through CNN. The long-term dependence features of PWC are retained through RNN. All extracted features are then combined linearly for classification.

Cardiovascular-disease classification experiments are performed on photoplethysmography (PPG) dataset and continuous non-invasive blood pressure (CNBP) dataset. Experimental results show that the classification accuracy of the multi-scale feature-extraction model can reach 80% and 96%, respectively.

The remaining part is organized as follows. In the ’Materials & Methods’ section, the experimental data are stated, and multi-scale feature-extraction model are proposed. In the ’Results’ section, the process of data pre-processing and experiment are stated in detail. Finally, conclusions are drawn in the ’Discussion’ section.

## Materials and methods

### Data description

At present, the most commonly used methods of collecting pulse wave signals information primarily include PPG and pressure detection. PPG traces the pulsation state of blood vessels and measures pulse wave signals by measuring the attenuated light reflected and absorbed by human blood vessels and tissues^27^. Pressure-detection method obtains pulse wave signals by directly detecting changes in the pressure of human arteries over time. The cuff is tied directly to the patient’s wrist, and the patient should maintain the same posture during measurement^28^. Patients feel that the wrist is sore and uncomfortable during long-term measurement. Compared with the pressure-detection method, the operation of PPG is simpler and suitable for long-term measurement, but it is susceptible to environmental interference during the measurement process, and its measurement accuracy and stability are lower than those of the pressure-detection method. For pulse wave signals obtained using different acquisition methods, to verify the generalization ability of the model, this study conducts experiments based on PPG dataset and CNBP dataset.

Considering that the clinically collected pulse wave signals contain substantial noise, pre-processing steps such as data filtering, beat division, etc. are necessary. The specific pre-processing details are described in the ’Results’ section.

- Dataset I: The PPG dataset for the non-invasive detection of cardiovascular diseases contains 657 pulse wave records from 219 subjects^29^. The data covers the age range of 20–89 years old, and the sampling rate is 1000 Hz. Various diseases including hypertension and diabetes are recorded. The dataset provides four labels for normal blood pressure, pre-hypertension, and stage I/II hypertension. Table 1 shows the details of dataset I.

**Table 1:** Dataset I label and sample size.

Type of disease	Number of samples(group)	Pre-processing tags	Number of samples(group)
Normal	80*3	Normal	593
Pre-hypertension	85*3	Hypertension	617
Hypertension Stage I	34*3		
Hypertension Stage II	20*3
- Dataset II: The CNBP dataset based on cardiovascular diseases is collected from the Fifth Affiliated Hospital of Zhengzhou University. The subjects who are 18–83 years old fill-up an informed-consent form before data collection. ZM-300 intelligent pulse wave signal collector is adopted for data acquisition, with a sampling frequency of 200 Hz. A pressure sensor based on a semiconductor strain gauge, with a sensitivity of 0.5 mV/g (bridge voltage of 6 V) and a pressure range of 0–1000 g, is used for the collection of pulse wave signals. The A/D converter is a 4-channel 12-bit converter. Dataset II contains 1326 pulse wave records from 221 subjects under six kinds of pulse pressures. It records various cardiovascular diseases, including hypertension and arrhythmia.
  Moreover, the dataset is collected under the control of standard experimental conditions and specifications. All methods are carried out in accordance with relevant guidelines and regulations, and all experimental protocols are approved by the Ethics Committee of Drug Clinical Trials of the Fifth Affiliated Hospital of Zhengzhou University. Table 2 shows the details of dataset II.

**Table 2:** Dataset II label and sample size.

Type of disease	Number of samples(group)	Pre-processing tags	Number of samples (group)
Normal	156*6	Normal	1582
Arrhythmia	18*6	Desease	1634
Myocardial ischemia	18*6		
Hypertension	15*6		
Others	14*6

### Multi-scale feature-extraction model

Few pulse wave datasets of cardiovascular diseases are available. At the same time, the feature division of PWC is unclear, and no uniform marking standard exists. The machine needs to learn PWC features automatically. In view of the above problems, a solution is to establish a feature-extraction model based on few-shot PWC. In this paper, a multi-scale feature-extraction model is proposed.

**Figure 1:**
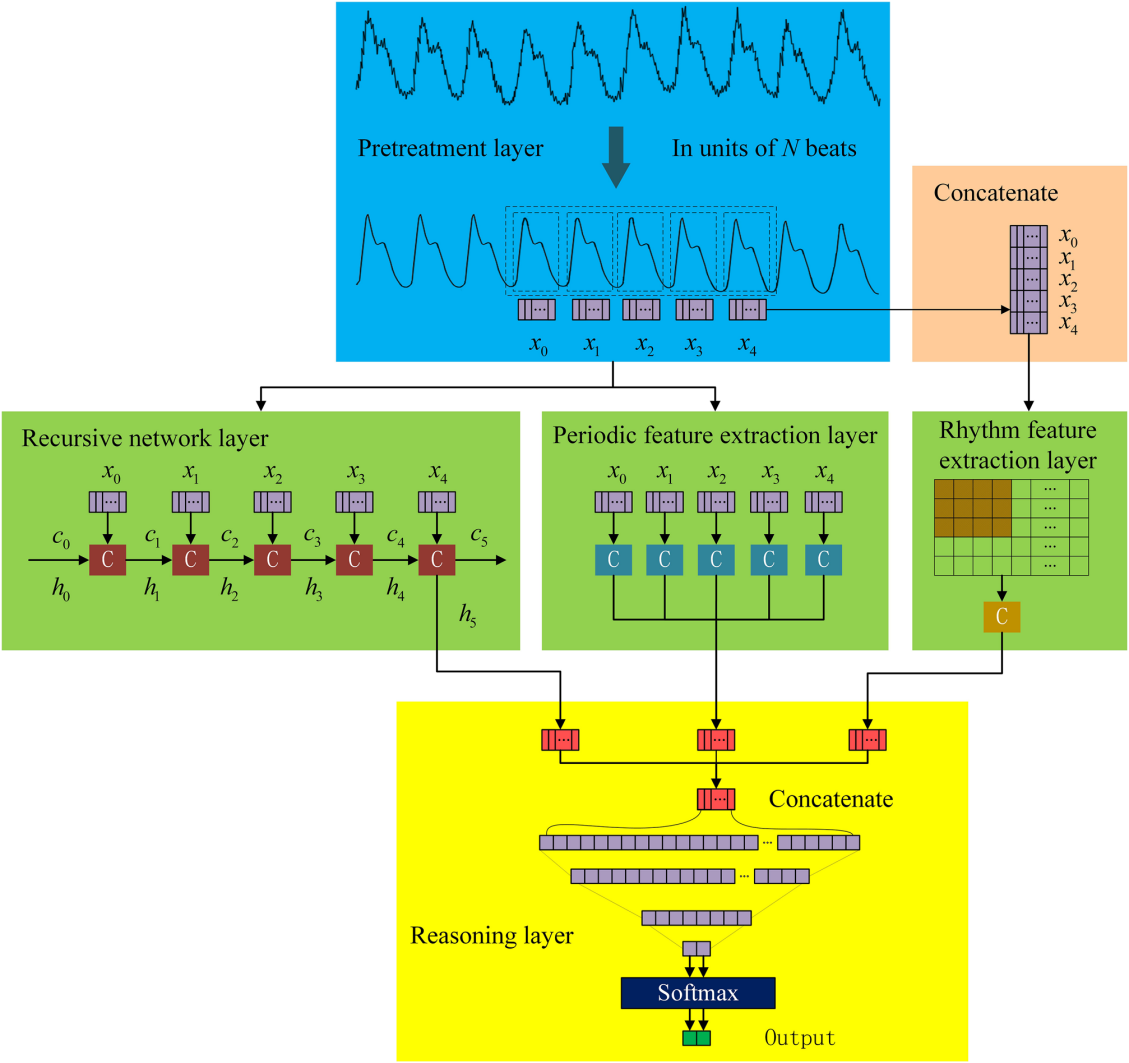
Network model structure.

The multi-scale feature-extraction model for few-shot PWC is shown in Fig. 1. *N* continuous PWC cycles are used as the network input, and then three network layers are connected in parallel to extract the multi-scale features of PWC. The recursive network layer is used to extract the length features of PWC; the periodic feature-extraction layer is used to extract the features of PWC within a single cycle; and the rhythm feature-extraction layer is used to extract PWC features between multiple cycles. Finally, features extracted from the three network layers are combined in the inference layer to classify PWC.

#### Recursive network layer

PWC is composed of multiple cycles of pulse fluctuations and belongs to a one-dimensional time series. RNN is a network which is good at dealing with time series. By modeling PWC through RNN, the correlation between several cycles of PWC can be obtained. PWC is segmented in chronological order and sequentially input into RNN. The output state of the hidden layer represents the long-term memory feature extracted as the recursive network layer.

**Figure 2:**
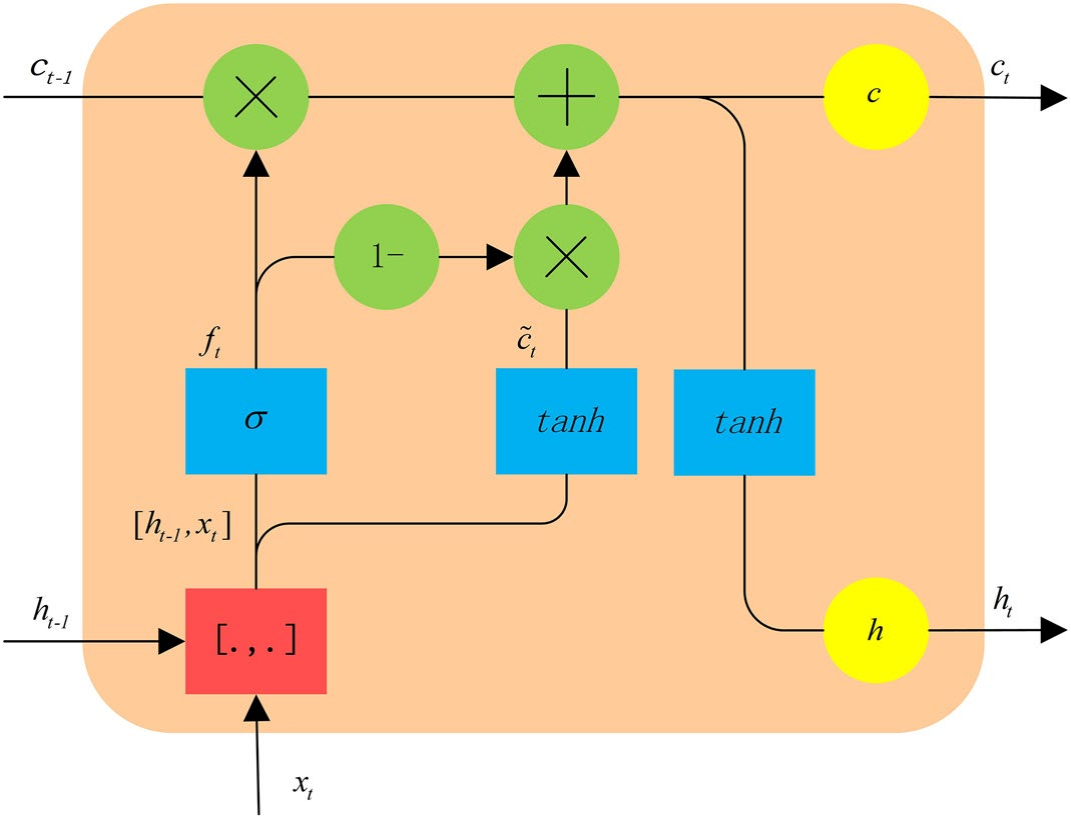
The unit structure.

Considering the number of sample points and the complexity of network training, a recursive-network unit structure for few-shot PWC is designed. The forgetting and saving of information at each moment are controlled by the forgetting gate. Compared with the LSTM unit structure, the parameters are fewer. The specific structure is shown in Fig. 2.

In the recursive-network unit for few-shot PWC, the unit state vector *c* is controlled by the forgetting gate. The number of unit states of the previous-cycle PWC retained in the current-cycle PWC is determined by the forgetting gate. The forward calculation formula of the forgetting gate is as follows:

1 $$\begin{aligned} ne{t_{f,t}}= & {} {W_f} \cdot [{h_{t - 1}},{x_t}] + {b_f} \end{aligned}$$

2 $$\begin{aligned} {f_t}= & {} \sigma (ne{t_{f,t}}) \end{aligned}$$

where the weighted input of the forgetting gate is represented by $ne{t_{f, t}}$, the weight matrix of the forgetting gate is represented by ${W_f}$, the operation of splicing two vectors into one vector is represented by $[{h_{t - 1}},{x_t}]$, the offset term of the forgetting gate is represented by ${b_f}$, the forgetting gate is represented by ${f_t}$, and $\sigma$ refers to sigmoid function.

The weight matrix ${W_f}$ is formed by splicing ${W_{fh}}$ and ${W_{fx}}$, corresponding to the previous-cycle PWC ${h_{t - 1}}$ and the current-cycle PWC ${h_t}$:

3 $$\begin{aligned} ne{t_{f,t}} = \left[ {\begin{array}{*{20}c} {W_{fh}}&{}{W_{fx}}\\ \end{array} } \right] \left[ {\begin{array}{*{20}c} {h_{t - 1}}\\ {x_t}\\ \end{array} } \right] + {b_f} = {W_{fh}}{h_{t - 1}} + {W_{fx}}{x_t} + {b_f} \end{aligned}$$

The input state ${{\tilde{c}}_t}$ of the current-cycle PWC is calculated from the unit output of the previous cycle and the input of the current cycle:

4 $$\begin{aligned} ne{t_{{\tilde{c}},}}_t= & {} {W_c} \times [{h_{t - 1}},{x_t}] + {b_c} = {W_{ch}}{h_{t - 1}} + {W_{cx}}{x_t} + {b_c} \end{aligned}$$

5 $$\begin{aligned} {{\tilde{c}}_t}= & {} tanh(ne{t_{{\tilde{c}},}}_t) \end{aligned}$$

where the weighted input of the PWC unit state is represented by $ne{t_{{\tilde{c}},t}}$; the weight matrix of the PWC unit state is represented by ${W_c}$, which is composed of two matrices ${W_{ch}}$ and ${W_{cx}}$; and the bias term of the forgetting gate is represented by ${b_c}$.

To update the cell state of the current cycle, the current memory ${{\tilde{c}}_t}$ needs to be combined with the long-term memory ${c_{t - 1}}$, and new information needs to be added whilst forgetting some information. The unit state ${c_{t - 1}}$ of the previous-cycle PWC is multiplied with the corresponding position element of the forgetting gate ${f_t}$, and the unit state ${{\tilde{c}}_t}$ of the current-cycle PWC is multiplied with the corresponding position element of $1 - {f_t}$ to obtain the state output of the current-cycle PWC ${c_t}$:

6 $$\begin{aligned} {c_t} = diag[{f_t}]{c_{t - 1}} + diag[1 - {f_t}]{{\tilde{c}}_t} \end{aligned}$$

The output is determined by the state of the PWC unit:

7 $$\begin{aligned} {h_t} = tanh({c_t}) \end{aligned}$$

**Figure 3:**
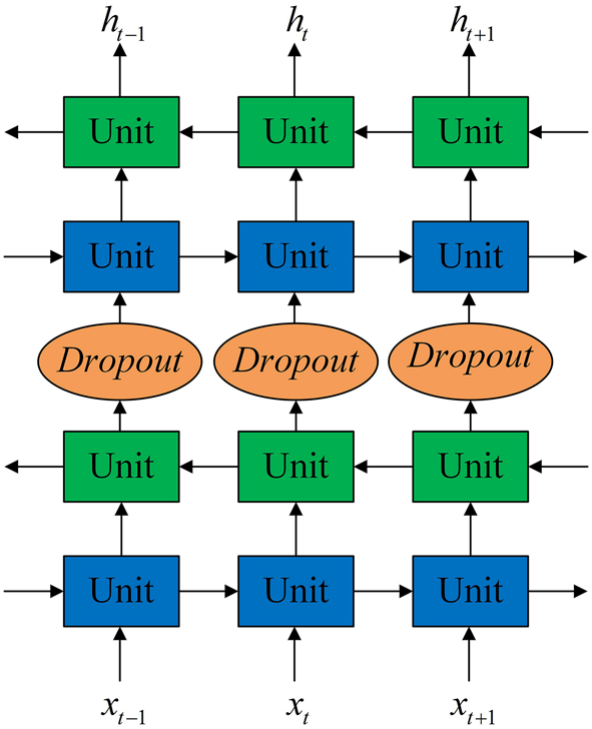
Recursive network layer structure.

As shown in Fig. 3, the recursive network layer is designed as a two-layer structure. The time of the main wave peak of the front PWC is used as the reference, and the single beat PWC of two adjacent troughs is used as the input of the unit. To mine the information that is opposite to the first-layer network-information transmission direction, a layer of reverse network is built on top of each layer of forward network, and the output of the first-layer network is directly used as the input of the reverse network. The output of the lower networks (${h_0}\sim {h_n}$) is used as the input of the reverse network after random screening by the upper layer:

8 $$\begin{aligned}&{r_i}\sim {Bernoulli(p)} \end{aligned}$$

9 $$\begin{aligned}&{x_t} = diag({r_t}) \times {h_t} \end{aligned}$$

where *Bernoulli*(*p*) represents the Bernoulli distribution according to probability *p* and has a value of 0 or 1; ${r_t} = [{r_0},{r_1},\ldots ,{r_n}]$, n are the dimensions of ${h_t}$, and ${x_t}$ is the input of the unit node at time *t* at the reverse network layer.

#### Periodic feature-extraction layer

The shape and change of PWC in a single cycle contain its main features. Extracting the spatial features of PWC in a single cycle is necessary. However, PWC is sparse and contains little information at a single moment. If all points of PWC are used as input sessions, the number of network parameters becomes too large. At the same time, the training efficiency and accuracy of the model are greatly reduced by too many features.

To effectively extract the features of PWC in a single cycle, one-dimensional CNN is used. The convolution module has the functions of local connection and weight sharing and can extract the spatial position relationship at different time points of a single-cycle PWC. At the same time, it can reduce the amount of network parameters and reduce the model complexity.

**Figure 4:**
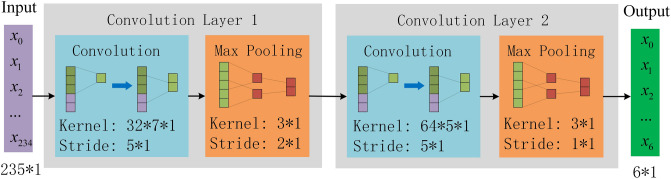
Structure of periodic feature extraction layer.

The single-cycle PWC is a signal segment with a length of 235, and it is used as the input. The specific structure of the single-period feature of PWC is obtained through two convolution modules, which each module containing a one-dimensional convolution layer and a maximum pooling layer. To reduce the amount of computation in the process of model training and improve the computation speed, ReLU function is used as the activation function. The specific structure is shown in Fig. 4. .

#### Rhythm feature-extraction layer

PWC is formed by the periodic and regular pulse of pressure in arteries. However, slight differences exist between PWC cycles, which contain rhythm information and are difficult to observe artificially. The rhythm features represent the feature relationship between cycles and reflects the envelope change of the pulse signal. These features have a classification effect and can improve classification accuracy. To extract the rhythm features of PWC, a two-dimensional CNN is used.

**Figure 5:**
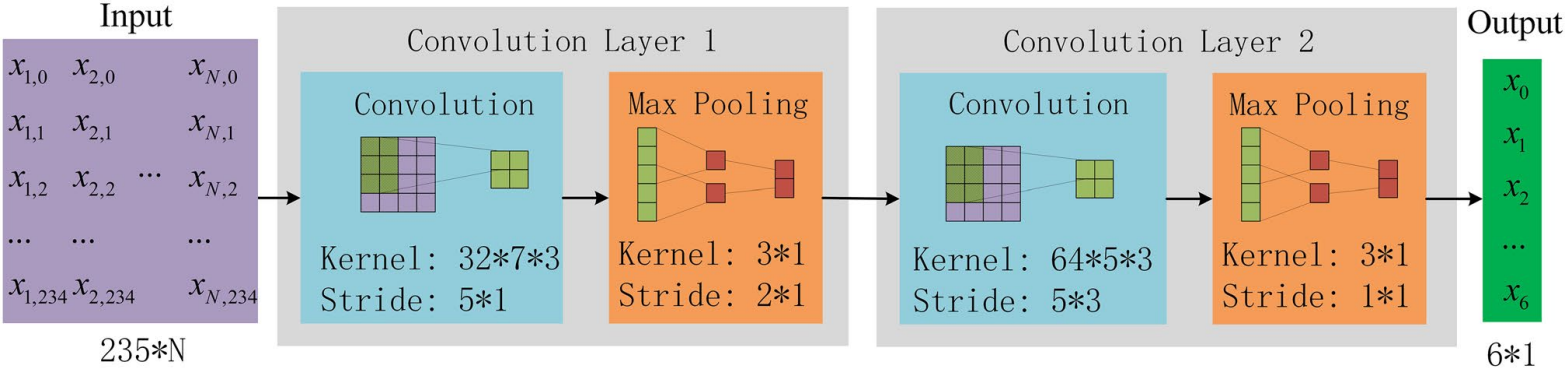
Structure of rhythm feature extraction layer.

Compared with single-cycle signals, multi-cycle signals have larger data dimensions, and a suitable convolution module scale is more important. Whilst ensuring that features are not discarded, reducing the dimension of data as much as possible is necessary. N adjacent periodic signals are spliced into a feature map, which is used as the input. The specific structure of the single-period features of PWC is obtained through two convolution modules, with each module containing one two-dimensional convolution layer and one max pooling layer. Same as the periodic feature-extraction layer, ReLU function is used as the activation function. The specific structure is shown in Figure 5.

#### Reasoning layer

The reasoning layer contains two fully connected layers, each neuron is fully connected to all neurons in the previous layer, and the local information with category discrimination in the network layer is integrated. The activation function of the fully connected layer adopts the ReLU function, and the training algorithm adopts the error back-propagation algorithm. The output of the last layer is passed through the SoftMax classifier to obtain PWC classification result.

Given that the gate-unit activation function in the recursive network layer is selected as the Sigmoid function, when the training result is close to the true value, the gradient operator is extremely small, and the convergence speed of the model is slow. The cross-entropy loss function is a logarithmic function. When it is close to the upper boundary, it can still be maintained at a high gradient state without affecting the convergence speed of the model. To improve the convergence speed of the model, cross-entropy is used as the loss function:

10 $$\begin{aligned} loss = - {1 \over N}\sum \limits _{i = 1}^N {\left( { - \sum \limits _{j = 1}^M {{y_{ij}}\log ({p_{ij}})} } \right) } \end{aligned}$$

where *N* is the number of samples, *M* is the number of PWC categories, ${y_{ij}}$ is the label of PWC, and ${p_{ij}}$ is the prediction result corresponding to each label.

## Results

### Evaluation index

We use Accuracy(Acc), Sensitivity (Sen), Specificity(Spe), Precision(Pre), and F1-score for model evaluation. The calculation formula for the five evaluation indicators is as follows^30^:

11 $$\begin{aligned} \mathrm{{Acc}}= & {} {{TP + TN} \over {TP + TN + FP + FN}} \end{aligned}$$

12 $$\begin{aligned} \mathrm{{ Sen}}= & {} {{TP} \over {TP + FN}} \end{aligned}$$

13 $$\begin{aligned} \mathrm{{Spe}}= & {} {{TN} \over {TN + FP}} \end{aligned}$$

14 $$\begin{aligned} \mathrm{{ Pre}}= & {} {{TP} \over {TP + FP}} \end{aligned}$$

15 $$\begin{aligned} F1 - score\mathrm{{ }}= & {} \mathrm{{ }}{{2 \times Pre \times Sen} \over {Pre + Sen}} \end{aligned}$$

where TP stands for true positive and is the number of samples predicting abnormal PWC; TN stands for true negative and is the number of samples predicting normal PWC as normal; FP stands for false positive and is the number of samples predicting normal PWC as abnormal; FN stands for false negative and is the number of samples predicting abnormal PWC as normal; Acc represents the overall classification accuracy of the overall model; Sen represents the proportion of abnormal PWC that are matched and measures the model’s ability to recognize abnormal PWC; Spe represents the proportion of normal PWC that are matched and measures the pairing of the classifier, i.e., the ability to recognize normal PWC; and Pre represents the proportion of PWC classified as abnormal that are actually marked as abnormal.

### Preprocessing

Pulse wave signals collected in clinical settings is easily affected by noise, including motion artifacts, inherent noise of collecting instruments, and power-supply noise. Using end-to-end training directly reduces the classification accuracy. Therefore, the original signals need to be pre-processed before the experiment.

The frequency of pulse wave signals is primarily distributed between 0.5–2 Hz. Motion artifact is primarily caused by breathing, and the respiratory frequency of normal adults is about 0.2–0.3 Hz. The inherent noise of collecting instruments is above 90 Hz. The power supply noise is 50 Hz/60 Hz. Empirical mode decomposition (EMD) is a signal-processing method based on the time-scale features of the data itself without pre-setting any basis functions^31^. EMD has obvious advantages in dealing with non-stationary and non-linear data. According to the frequency-distribution features of the pulse wave signals, the EMD method is used to remove the noise.

In the process of EMD, cubic spline function is used to interpolate the maximum value sequence and the minimum value sequence to obtain the upper and lower envelopes. The cubic spline interpolation needs to use two adjacent points before and after. Therefore, there will be divergence at the two ends of the data, that is, the derivative of the intrinsic mode-function at the boundary increases, which makes the filtered signal has obvious distortion at the beginning and end. To avoid the transient phenomenon at the beginning and end of the filtered signal, the end points of the curve are added to the spline. The Pearson correlation degree is used to measure the degree of information loss before and after denoising, and the calculation method is shown in equation (16):

16 $$\begin{aligned} Pearson = {{\sum {XY} - {{\sum X \sum Y } \over N}} \over {\sqrt{\left( {\sum {{X^2}} - {{{{(\sum X )}^2}} \over N}} \right) \left( {\sum {{Y^2}} - {{{{(\sum Y )}^2}} \over N}} \right) } }} \end{aligned}$$

where *X* represents the set of elements arranged in time in the original PWC, *Y* represents the set of elements arranged in time after denoising, and *N* represents the number of sample points.

**Figure 6:**
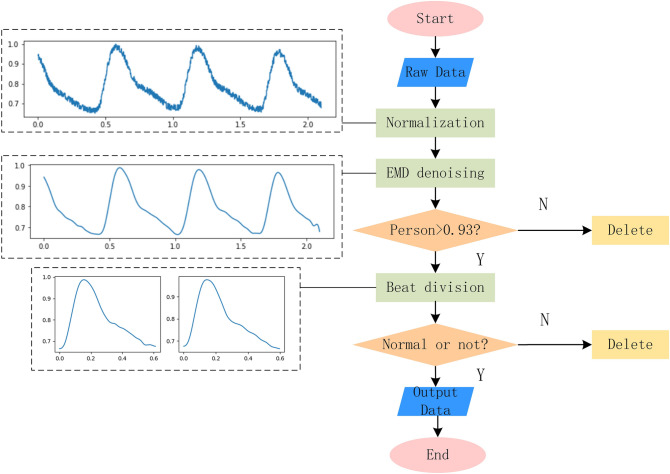
Network model structure.

The value interval of Person is [− 1,1]. When a person is close to 1, *X* and *Y* have a strong linear correlation, showing that the information loss is relatively small after denoising. In the experiment, only data with a Person coefficient greater than 0.93 after filtering is retained to ensure that the filtered data retains the original information to the greatest extent. The calculation result of the signal Person coefficient in Fig. 6 is 0.99.

Signals collected clinically are actually the slice data of pulse wave signals. Few records of pulse wave database itself exist; not every record has all categories, and even some categories appear only on a few records. In this case, to ensure that categories are not omitted as far as possible, the PWC should be divided separately into beats^32^. In the experiment, the PWC is divided into cycles according to the trough position. The signal segment of the adjacent valleys is regarded as one PWC cycle. The specific pre-treatment process is shown in Fig. 6.

### Setting of model parameters

The pre-processed PWC is randomly divided into training set and test set at a ratio of 4:1. Under the condition of a certain amount of data, the classification accuracy of the model no longer increases before the number of hidden nodes of the neural network increases to a certain extent^33^. Therefore, the number of hidden nodes in the recursive network layer is set to 32, the number of kernels in the periodic feature-extraction layer is set to 32 and 64, the number of kernels in the rhythm feature-extraction layer is set to 32 and 64. After verification through multiple experiments, the network training parameters are set, as shown in Table 3.

**Table 3:** Network hyperparameters.

Parameter		Value	
Periodic feature-extraction layer		Convolution Layer 1	Convolution Layer 2
	Kernel dimension	7*1	5*1
	Stride size	5*1	5*1
	Amount of kernels	32	64
	Pooling dimension	3*1	3*1
	Pooling stride size	2*1	1*1
Rhythm feature-extraction layer	kernel dimension	7*3	5*3
	Stride size	5*1	5*3
	Amount of kernels	32	64
	Pooling dimension	3*1	3*1
	Pooling stride size	2*1	1*1
Recursive network layer	Input channels	235	
	Number of hidden nodes	32	
	Bidirectional	True	
	Dropout	0.5	
Cycle length of PWC		235	
Unit cycle		N	
Reasoning layers		Feature dimension*32/32*2	
Batch size		64	
Optimizer		Adam	

### Experiment

The number of PWC cycles input of the model is expressed as a unit cycle. An increase in amount of cycles of model input leads to an increase in depth of the recursive network. Furthermore, the proportion of features extracted by the periodic feature-extraction layer and the rhythm feature-extraction layer increase. At the same time, the network dimension increases leads to decreased training speed of the model. To determine the optimal number of unit cycles for a suitable PWC multi-feature scale model, experiments are conducted for different unit cycles. Training set II is used in the experiment, and each group is trained 50 times. The final results are shown in Table 4. According to the table, when the number of unit cycles is 5, the model has the optimum effect.

**Table 4:** The influence of different unit cycles on experimental results.

Unit cycles	Acc(%)	Sen(%)	Spe(%)	Pre(%)	F1
2	93.17	93.25	92.50	92.68	92.97
3	94.35	94.86	92.96	93.12	93.98
4	95.24	93.37	96.03	96.27	94.80
5	**96**.**39**	**94**.**66**	**96**.**67**	**96**.**88**	**95**.**75**
6	94.34	91.74	93.94	94.34	93.02

**Figure 7:**
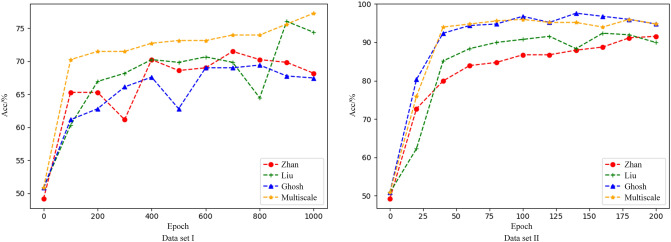
Changes in classification accuracy of different network training processes.

On the premise of retaining the structure of the pre-processing layer and the reasoning layer, as well as the network parameters, the network layer is changed for comparison experiments. Zhan^34^ et al. extracted features through CNN for classification. Liu^35^ et al. extracted PWC timing features through parallel CNN structures, including signal segment features within a period and multi-period features representing cycle relationships; Ghosh^36^ et al. extracted PWC features based on LSTM to predict systolic and diastolic blood pressure. The training process of different network models is shown in Figure 7.

Performance comparison with other neural network methods under different datasets is shown in Table 5. According to the table, the multi-scale model has good classification performance on dataset I and dataset II.

**Table 5:** Performance comparison of multi-scale model and other methods.

Model	Dataset I (PPG)		Dataset II (CNBP)	
	Acc(%)	F1	Acc(%)	F1
Zhan	76.02	74.82	92.71	92.12
Liu	78.16	78.33	91.94	91.44
Ghosh	72.54	67.54	95.72	95.35
Multi-scale feature extraction model	**79**.**75**	**79**.**01**	**96**.**22**	**95**.**81**

## Discussion

A small-parameter unit structure and a multi-scale feature-extraction model are proposed to obtain improved results under the condition of few-shot pulse wave contour classification. Cardiovascular-disease classification experiments were carried out on PPG dataset and CNBP dataset. To evaluate the classification performance of the multi-scale feature-extraction model, three different classification methods including single neural network model^34^, parallel neural network model^35^, and LSTM model^36^ were introduced for comparison. Comparative studies show that the multi-scale feature-extraction model outperforms the other classification methods in terms of identification accuracy, stability, and sensitivity, and the multi-scale feature-extraction model consumes less time for training. For the proposed novel PWC classification approach, the model is notably sensitive to the number of unit cycles, and we find that the best unit cycle is five. Also, the multi-scale feature-extraction model depends on the division result of the PWC cycle.

Moreover, (a) the classification problem of few-shot PWC is well handled; (b) owing to the limitation of data volume, only binary classification is carried out; (c) PWC must be pre-processed (including EMD filtering and beat segmentation) before feature learning, which may lead to missing partial information. Finally, given the excellent performance of the multi-scale feature-extraction model in the few-shot PWC experiments, the classification of few-shot PWC is interesting and meaningful to study, and it will be further investigated in our future research.
